# Diversity and composition of herbaceous angiosperms along gradients of elevation and forest-use intensity

**DOI:** 10.1371/journal.pone.0182893

**Published:** 2017-08-08

**Authors:** Jorge Antonio Gómez-Díaz, Thorsten Krömer, Holger Kreft, Gerhard Gerold, César Isidro Carvajal-Hernández, Felix Heitkamp

**Affiliations:** 1 Section of Physical Geography, Georg-August-Universität Göttingen, Göttingen, Germany; 2 Centro de Investigaciones Tropicales, Universidad Veracruzana, Xalapa, Veracruz, Mexico; 3 Department of Biodiversity, Macroecology & Biogeography, Georg-August-Universität Göttingen, Göttingen, Germany; 4 Herbario CIB, Instituto de Investigaciones Biológicas, Universidad Veracruzana, Xalapa, Veracruz, Mexico; Chinese Academy of Forestry, CHINA

## Abstract

Terrestrial herbs are important elements of tropical forests; however, there is a lack of research on their diversity patterns and how they respond to different intensities of forest-use. The aim of this study was to analyze the diversity of herbaceous angiosperms along gradients of elevation (50 m to 3500 m) and forest-use intensity on the eastern slopes of the *Cofre de Perote*, Veracruz, Mexico. We recorded the occurrence of all herbaceous angiosperm species within 120 plots of 20 m x 20 m each. The plots were located at eight study locations separated by ~500 m in elevation and within three different habitats that differ in forest-use intensity: old-growth, degraded, and secondary forest. We analyzed species richness and floristic composition of herb communities among different elevations and habitats. Of the 264 plant species recorded, 31 are endemic to Mexico. Both α- and γ-diversity display a hump-shaped relation to elevation peaking at 2500 m and 3000 m, respectively. The relative contribution of between-habitat β-diversity to γ-diversity also showed a unimodal hump whereas within-habitat β-diversity declined with elevation. Forest-use intensity did not affect α-diversity, but β-diversity was high between old-growth and secondary forests. Overall, γ-diversity peaked at 2500 m (72 species), driven mainly by high within- and among-habitat β-diversity. We infer that this belt is highly sensitive to anthropogenic disturbance and forest-use intensification. At 3100 m, high γ-diversity (50 species) was driven by high α- and within-habitat β-diversity. There, losing a specific forest area might be compensated if similar assemblages occur in nearby areas. The high β-diversity and endemism suggest that mixes of different habitats are needed to sustain high γ-richness of terrestrial herbs along this elevational gradient.

## Introduction

The majority of biomes are undergoing rapid changes, especially in the tropics [1]. Consequently, growing human pressure on ecosystems poses a marked threat to global biodiversity [2]. Considering current rates of deforestation and forest degradation [3], undisturbed forests will become scarce and increasingly fragmented [4]. Deforestation for agriculture, as well as non-sustainable agrarian and forestry practices, increases the demand for new land threatening primary forests and associated biodiversity [5]. Forest-use intensity is defined as a forest disturbance derived from the human action acting at a broader landscape level. The effects of forest conversion on plant diversity are well known [6]; however, there is a lack of knowledge on how anthropogenic forest-use intensity affects diversity and composition of plant communities [7]. Forest degradation may have different effects on biodiversity, depending on the ecosystem, the kind of degradation (temporal and spatial extent, intensity) and the taxa of interest [6].

A global meta-analysis of trends of forest degradation found an average loss of 18% of species richness due to forest use [8]. According to the analysis, land-use change and the increase of non-native invasive species have the highest negative impact on species richness [8]. However, some landscapes have experienced increases in plant species richness because invasions of exotic species are exceeding the loss of native species [9]. The magnitude of anthropogenic change and high diversity in tropical mountains calls for more studies to identify the patterns of diversity change due to forest-use intensity along elevational gradients [10].

Little is known about the influence of forest-use intensity on herbaceous angiosperms despite their importance for tropical diversity and ecosystem function [11]. The few studies available have mixed results with forest-use intensity reported to have positive, neutral, or negative effects [12]. Moreover, high numbers of primary forest species and endemic species have been found in naturally regenerating [13] and secondary forests. Thus, such habitats might help to conserve endemic species [14]. Forest use may also lead to an increase in species numbers due to an alteration of the light regime and a suppression of more competitive herbs [15]. Contrasting findings may reflect different study conditions (biome, ecosystem, taxa of interest), different scales (e.g. landscape, plot) and sampling techniques involved [16]. Therefore, it is important to carry out more empirical research to quantify the effects of forest-use intensity on herbs using a robust and replicated study design that accounts for the different components of herb diversity (α, β, and γ).

Despite the uncertainties about forest-use intensity effects on species richness, changes in species composition have been reported often with the rarest species found mainly in native communities [17]. Several studies have shown that with increasing forest-use intensity, local (*α*-diversity) and total (*γ*-diversity) species richness declined linearly, whereas species turnover between plots increased [18]. The forest-use intensity in the most intensively used plots led to sparse herb layer and reduced species richness [19].

Studies on latitudinal and elevational gradients show that effects of forest-use intensity on plant diversity may change depending on the ecosystem or ecozone. In general, α- and γ-diversity of plants decrease with increasing latitude, [20,21], and the highest diversity of plants and various others taxa is usually concentrated in tropical lowland areas with high and evenly distributed rainfall [22,23]. Several studies of tropical elevational gradients have shown mid-elevations peaks in species diversity followed by a linear decrease [20] for various plant groups [24]. Most of the previous studies on elevational gradients of forest herbs focused on selected herbaceous families [25] and were conducted in near-natural ecosystems. Given the ongoing degradation of primary forests [6], studies on elevational gradients should be extended to include habitats that differ in forest-use intensity or anthropogenic influence.

This study aims to assess how herbaceous angiosperm diversity varies along elevational gradients in the tropics and how those patterns are affected by variation in forest use intensity. We thus focused on patterns of α-, β-, and γ-diversity of herbaceous angiosperms along combined gradients of elevation and forest-use intensity. The study was conducted along the Eastern slopes of the volcano *Cofre de Perote* in central Veracruz, Mexico, along an elevational gradient from sea level up to 3500 m, which exhibits a large range of environmental conditions over a short geographic distance of just *c*. 80 km. We established plots at eight different locations (separated by *c*. 500 m in elevation) and with three different forest-use intensities (old-growth, degraded, and secondary forest).

## Methods

### Study area

**Fig 1:**
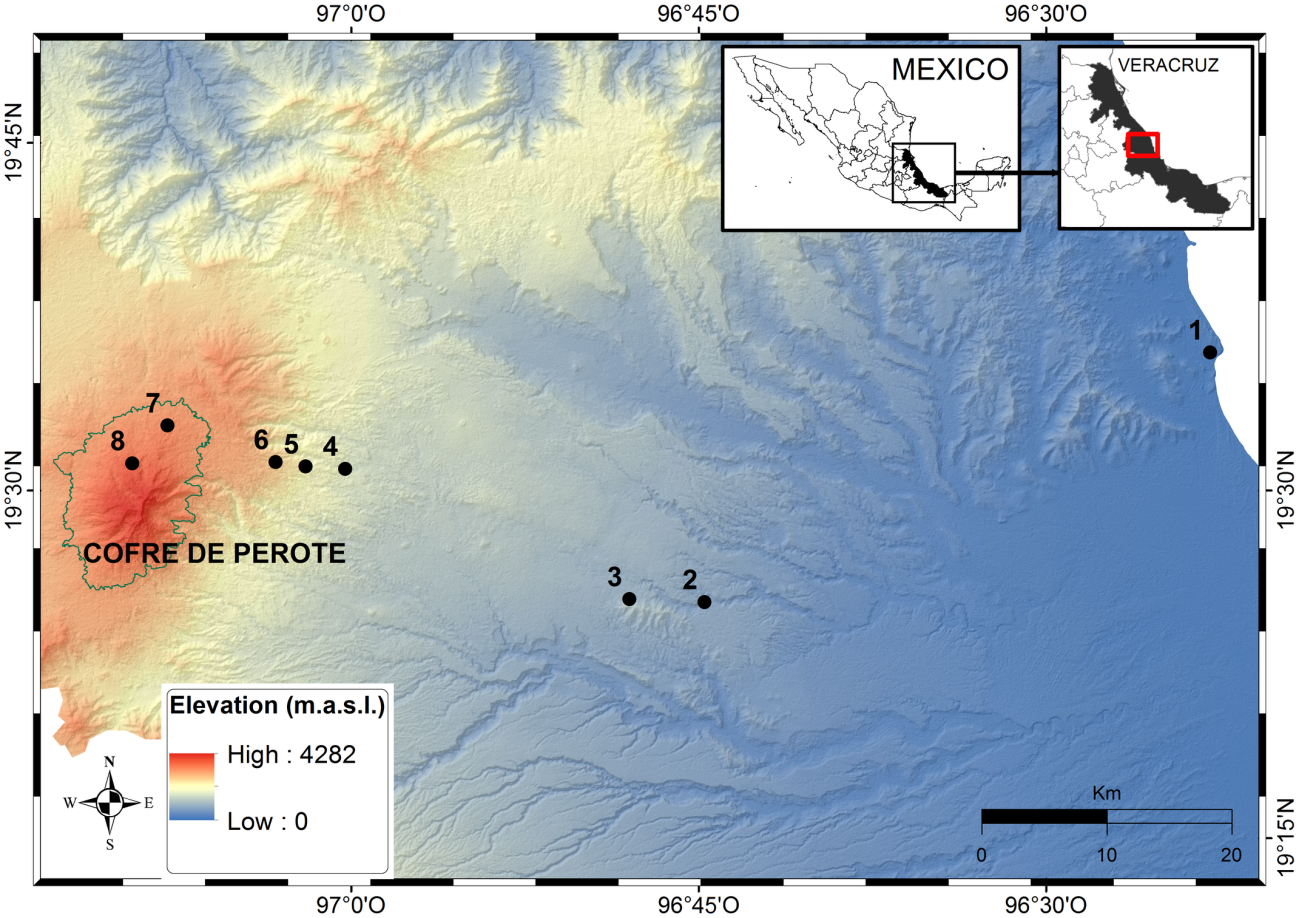
Map of the Eastern slopes of the *Cofre de Perote* in Veracruz state, Mexico. Black dots show the study locations. 1 = *La Mancha*, 2 = *Palmarejo*, 3 = *Chavarrillo*, 4 = *Los Capulines*, 5 = *El Zapotal*, 6 = *El Encinal*, 7 = *Los Pescados*, and 8 = *El Conejo*.

We established eight sites along an elevational gradient between 30 and 3540 m on the Eastern slopes of the National Park *Cofre de Perote*, an extinct volcano of 4282 m elevation in the central part of the state of Veracruz, Mexico (Fig 1). This region is located at the junction of the Trans-Mexican volcanic belt and the *Sierra Madre Oriental*, a mountainous area between 19° 25’ 5.7” and 19° 36’ 54” N and 94° 44’ 43.5” and 97° 09’ 36.9” W. The state of Veracruz covers 72420 km^2^ and has a diverse angiosperm flora (6876 species), which represents about 31% of the Mexican flora [26]. More than 80% of Veracruz’ primary vegetation has been converted and the remaining parts are highly fragmented [27], therefore it is recognised as a priority region for conservation in Mexico [28].

We placed our study locations at the following elevations above sea level: 30–50 m, 610–670 m, 900–1010 m, 1470–1650 m, 2020–2230 m, 2470–2600 m, 3070–3160 m and 3480–3540 m (Table 1, Fig 1). To simplify, from now on we will refer to every site as a categorical unit (50, 650, 1000, 1500, 2100, 2500, 3100, 3500 m). We used a Garmin^®^ GPSMAP 60Cx device to record information about geographical reference and elevation.

**Table 1:** List of the study locations and climatic conditions along the elevational gradient at the *Cofre de Perote*, central Veracruz, Mexico. Information is given on elevational range, vegetation type according to Leopold [29], mean annual temperature (MAT), mean annual precipitation (MAP), days of rain (DR), and days below 0°C according to National Meteorological Service of Mexico (data from 1951–2010) [30].

Location	Elevation (m)	Vegetation type	MAT (°C)	MAP (mm)	DR	DB
*La Mancha*	30–50	Tropical semi-humid deciduous forest	26	1221	81	0
*Palmarejo*	610–670	Tropical semi-humid deciduous forest	23	938	86	0
*Chavarrillo*	900–1010	Tropical *Quercus* forest	21	1552	123	0
*Los Capulines*	1470–1650	Humid montane forest	18	1598	145	0
*El Zapotal*	2020–2230	Humid montane forest	14	3004	199	3
*El Encinal*	2470–2600	*Pinus*-*Quercus* forest	12	1142	100	12
*Los Pescados*	3070–3160	*Pinus* forest	10	821	113	14
*El Conejo*	3480–3540	*Abies* forest	8	829	112	16

### Data collection

**Fig 2:**
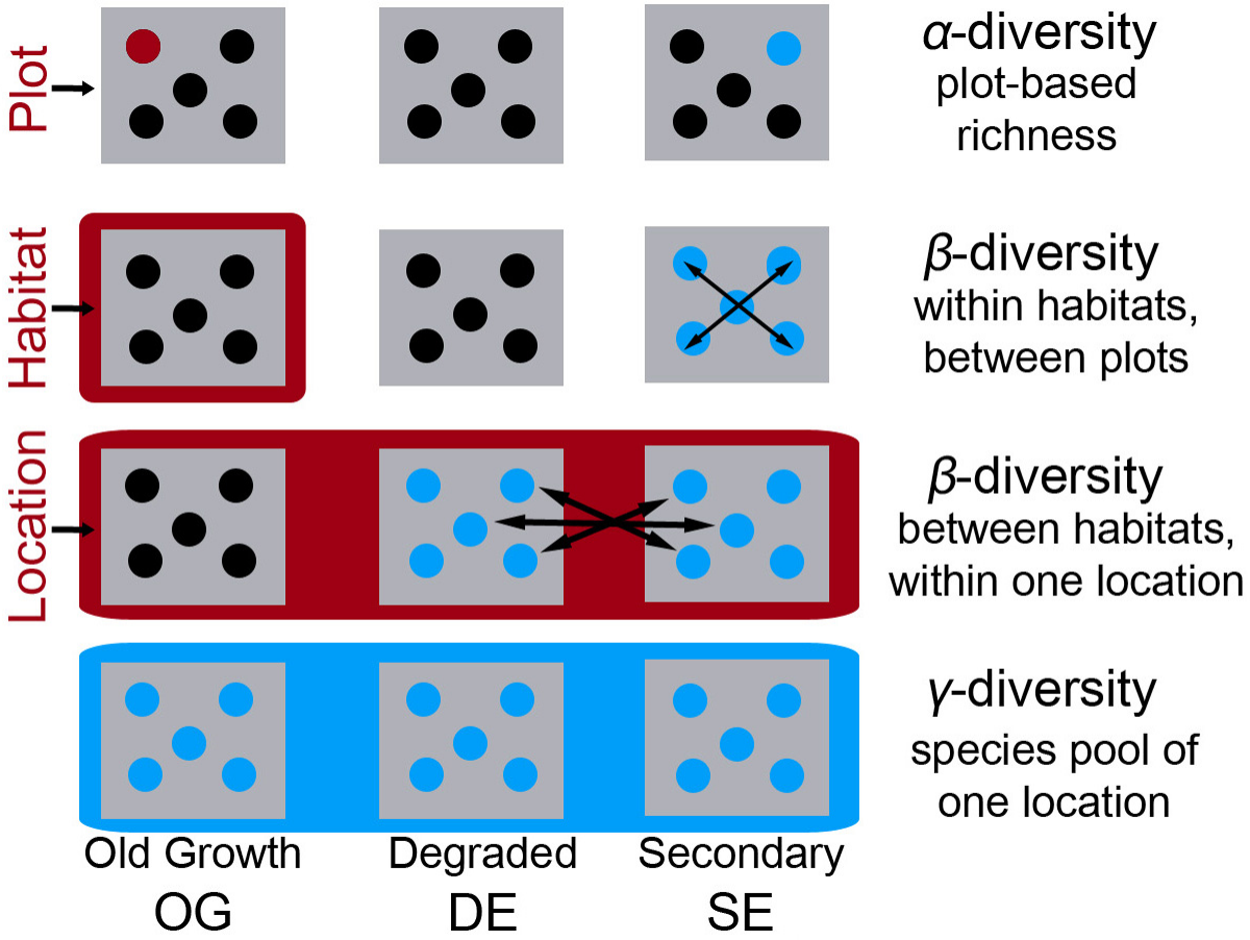
Schematic representation of the sampling design. We measured α-diversity in plots of 20 m x 20 m given as a mean of five plots. Five plots represent one habitat. We defined habitat as a homogenous type of forest-use intensity within one location. A location is representative of an elevational belt and harbors three different habitats (old-growth OG, degraded DE, and secondary SE). We measured two different β-diversities based on pairs of plots. Within-habitat β-diversity represents the compositional heterogeneity of a habitat. It is measured as the 1-Sørensen index based on multiple pairwise comparisons of the five plots within each habitat of a specific location. Between-habit β-diversity represents the compositional heterogeneity between different forest-use intensity. Measurement is similar to within-habitat β-diversity, but multiple pairwise comparisons based on the plots between habitats of a specific location. We defined γ-diversity as the total number of the local species pool across all 15 plots within a location, i.e. three habitats with five plots each.

The sampling design, as well as frequently used terms are shown in Fig 2. Fieldwork was conducted between February 2012 and January 2014. We sampled the presence/absence of terrestrial herbaceous angiosperms, which we defined as plants without a persistent aboveground woody stem or plants with only slightly woody stems, rooted on the forest floor and of short stature (generally < 1 m); vines were excluded [31]. We recorded all species in 20 m × 20 m plots, without considering seedlings [32]. We choose a plot size of 400 m^2^ because for the herbaceous flora in humid tropical forests this area is regarded to be representative, while small enough to minimise within-plot variation of abiotic factors [33,34]. We located the plots in the three different habitats subjected to different degrees of forest-use intensity; old-growth (OG), degraded (DE), and secondary forest (SE) stands (n = 5 plots for each habitat). These categories follow Newbold et al. [35] and are defined in Table 2. Each habitat was present at each of the eight locations resulting in a total number of 120 plots and a sampled area of 48000 m^2^. The Secretaría de Medio Ambiente y Recursos Naturales (SEMARNAT SGPA/DGVS/2405/14) issued us a plant collection permit (NOM-059-SEMARNAT-2010), which covered the whole study area and the collection of protected species mentioned in the Mexican legislation.

**Table 2:** Classification of habitats with different forest-use intensities according to the main physiognomic characteristic, the gap fraction in the canopy, dominance of canopy trees, the percentage of shrubs, and the presence of lianas [35].

Habitat	Characteristic	Gaps (%)	Forest-use intensity	Canopy trees	Shrub (%)	Lianas
Old-growth	No obvious forest-use, dominance of mature trees	<10	Low	High	<30	No
Degraded	Selective logging, grazing and understory removal	11–25	Medium	Low	30–50	Low
Secondary	Regrown after clear-cut	>25	High	very low	>50	High

We used elevation (m), mean annual temperature (°C), mean annual precipitation (mm/a) and habitat (a factor with three levels OG, DE and SE), as well as their interaction as explanatory variables. We obtained mean annual temperature (MAT) using data loggers (HOBO PRO v2) for one year (January-December 2014). We installed a total of 42 data loggers, two in every forest-use habitat at six elevations (500 m, 1000 m, 1500 m, 2500 m, 3000 m, and 3500 m) along the entire gradient. Within the plots, we placed data loggers on trees at a height between two and three meters. We obtained mean annual temperature data for the two elevations (50 m and 2000 m) and data of the mean annual precipitation (MAP) from eight climatological stations (near to the sampling sites) operating along the elevational gradient during the period 1951–2010 [30].

### Species identification

We collected at each location, but not in every plot, specimens of all species if possible in quadruplicates and deposited at the Mexican herbaria CIIDIR, MEXU, XAL, and XALU. Details about species identifications, geographical distribution and classification can be found in Gómez-Díaz et al. [36]. The presence/absence data of all the species found at the plots can be found at the Supporting Information (S1 File).

### Data analyses

We calculated for each of the 120 herb communities in the dataset, i.e. replicated plots (5 plots per habitat x 8 locations = 120), α-diversity, the proportion of endemics and β-diversity [37]. Alpha-diversity is the count of the number of species (plot-based species richness) in each standardised replicate plot [38]. In order to evaluate the value of endemic species, we measured the proportion of endemic species per plot. We used the dissimilarity (1-S) variant of the Sørensen index [37] as a measure of β-diversity and we calculated it as:

β−diversity=1−S (1)

where S is the Sørensen index:

$$S=\frac{2C}{A+B}$$ (2)

which is a coefficient of association, where a value of 1 shows that a pair of plots under consideration has exactly the same species. The Sørensen index can be adjusted to measure species turnover (effective or real) [39]. The index 1-S is also a measure of β-diversity because if a coefficient is near to 1, it shows that the units do not share species, and, therefore, they have high β-diversity [40]. As a measure for landscape-scale β-diversity, we compared the effect of habitats on floristic composition between OG and DE, OG and SE as well as DE and SE at each site [41]. We calculated plot-by-plot, i.e. each plot of habitat A was compared to five or four (in the case of within β-diversity) other plots of habitat B. Additionally, we calculated the standard errors for the β-diversity estimator, letting a statistically rigorous contrast of two or other similarity index values. Standard errors were calculated by the bootstrap process, which needs resampling the observed data for pairs of samples and re-computing the estimators *N* times [41]. We performed these analyses of β-diversity in EstimateS 9.1.0 [42].

We used a set of generalized linear models (GLM) and informatic theoretic approach based on the bias-corrected Akaike’s information criterion (AIC_c_) to examine the extent to which herb α-diversity, the proportion of endemics and β-diversity is related to elevation, habitat, MAT and MAP. These sets of models aimed to describe the pattern of distribution of the response variables, namely herb α-diversity, the proportion of endemics and β-diversity. We used elevation, elevation^2^, habitat, MAT, MAT^2^, MAP, MAP^2^ and various two-way and three-way interactions as explanatory variables in these models. We included polynomial terms of elevation, MAT and MAP in order to detect potential hump shapes in the relationships between elevation and the inter-correlated climatic variables with the response variables. We did not include the data below 500 m in the case of β-diversity due to the lack of species in six plots and we used “habitat transition” with six levels (OG to OG, OG to DE, OG to SE, DE to DE, DE to SE and SE to SE) instead of the independent variable “habitat”. We constructed thirty-one plausible models combining these variables for this set of models [38].

We checked data for normality using the Shapiro-Wilk normality test. We checked the homogeneity of variance using the Bartlett test. None of the variables followed a normal distribution. Therefore, we chose an error structure for each variable depending on the nature of the variable. We choose the error structure according to the “descdist” function of the R package “fitdistrplus” version 1.0–7. We used a negative binomial error structure for models of α-diversity. We used a beta error structure for models of the proportion of endemics. We used a log-normal error structure for models of β-diversity. We used maximum likelihood estimation and AIC_c_ values compared to choose the best model for each response variable in each set of models. We performed all analyses in R 3.2.1 [43]. Finally, we calculated an *R*^2^ for each model [38].

#### Multiplicative diversity partitioning

We used multiplicative diversity partitioning [44] and focused on three distinct components of community differentiation (α-diversity), (β-within habitat) and compositional dissimilarity (β-between habitats) [45,46]. We partitioned total γ-diversity per location into multiplicative components representing per plot α-diversity, within habitats β-diversity and among habitats β-diversity. We used the function “multipart” of the R package “vegan” to partition diversity [45,47]. Through the text, we will refer to the results of this analysis as relative (relative α-diversity, β-within habitat, etc.)

#### Estimation of species diversity (Hill numbers)

Following Chao and Jost [48], we used a diversity profile estimator of ^*q*^*D* (Hill numbers). The diversity estimator of order *q* in each profile represents the asymptote in the rarefaction and extrapolation curves [49]. We calculated the profile estimator with the function “diversity” of the R package “SpadeR” [50]. We considered the cases of *q* from 0 to 3 with increments of 0.25.

## Results and discussion

### Alpha diversity

**Fig 3:**
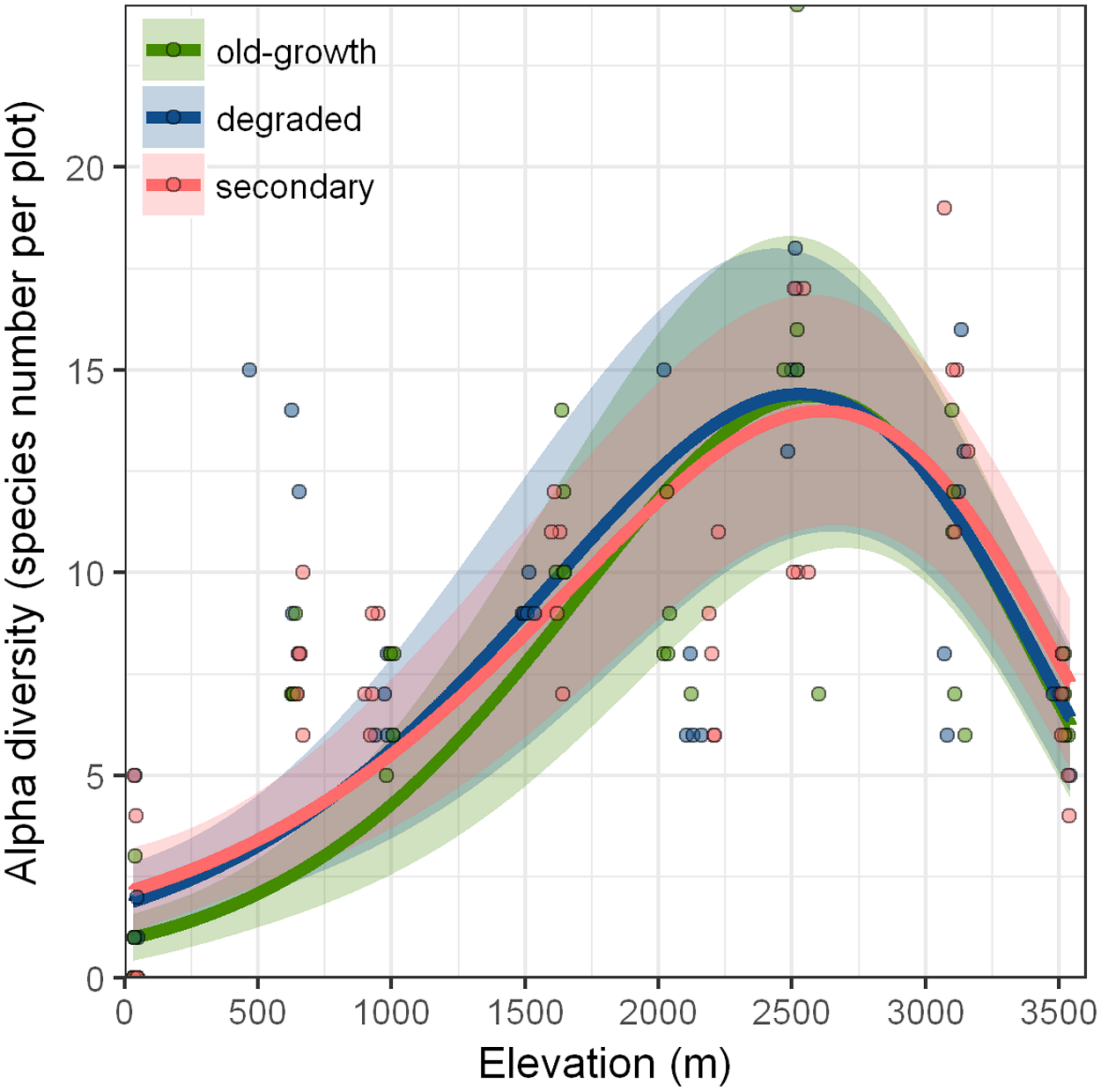
Alpha-diversity of herbaceous angiosperms along gradients of elevation and forest-use intensity at the *Cofre de Perote*, central Veracruz, Mexico. We fit the lines from a negative generalized linear model (GLM), the shaded area marks confidence intervals (CI = 1.96 times standard error). Difference to zero is significant for the intercept (xy, OG at 50 m, p < 0.001), but it is not significant for the effect of habitat (DE: p = 0.453, SE: p = 0.446). Observed species richness on 20 m × 20 m plots along the elevational gradient for OG (green), DE (blue), and SE (red).

Elevation, elevation^2^, habitat, MAT, MAP^2^, interactions between elevation and elevation^2^, an interaction between elevation and MAT, as well as an interaction between elevation^2^ and MAT modeled best the α-diversity (Table 3). This model explained a large amount of the variance in species richness (GLM *R*^2^ = 0.69). Alpha-diversity followed a hump-shaped pattern, showing a peak at 2500 m and declines towards the extremes of the gradient that do not change depending on the habitat (Fig 3, Table 4). Fewer species are found at high MAT and this effect is greatest at low elevations due to the inclusion of the MAT + elevation interaction.

**Table 3:** Summaries of generalized linear models. The summaries link herb α-diversity, the proportion of endemics and β-diversity to environmental explanatory variables, along an elevational gradient at central Veracruz, Mexico. We reported the best models, according to the bias-corrected Akaike information criterion (AICc). We also give the change in AICc between the best model and the next best and worst. Finally, an R^2^ measuring variation explained by the model is given.

Response	Model	AIC_c_	ΔAIC_c_ (next best)	ΔAIC_c_ (worst)	*R*^2^
α-diversity	Elevation + elevation^2^ + habitat + MAT + MAP^2^ + elevation:MAT + elevation:elevation^2^ + MAP:elevation^2^	570.96	0.1	1023.3	0.69
the proportion of endemics	Elevation + elevation^2^ + habitat + MAT + MAP^2^ + elevation:MAT + elevation:elevation^2^ + elevation:MAT^2^ + elevation:elevation^2^:MAT	-162.50	1.9	111.7	0.41
β-diversity	Elevation + elevation^2^ + change + MAT + MAP + elevation:change + elevation:MAT + elevation^2^:change + elevation^2^:MAT + change:MAT + MAT:MAP + elevation:change:MAT + elevation^2^:change:MAT	287.41	1.7	450.6	0.49

**Table 4:** Parameter estimates from generalized linear models. The parameters link herb α-diversity, the proportion of endemics and β-diversity to environmental explanatory variables, along an elevational gradient at central Veracruz, Mexico. Estimates are on the standardized scale ± standard error. We marked significant estimates with an asterisk (* < = 0.05, ** < = 0.01 and *** < 0.001). Empty cells indicate terms not included in the best model for a given response variable.

	Estimates		
Term	α-diversity	the proportion of endemics	β-diversity
Elevation	-5.66 ± 0.04***	7.22 ± 1.25	-2.89 ± 0.38***
Elevation^2^	7.09 ± 0.28**	-5.74 ± 1.72	1.98 ± 0.24***
Habitat.DE	0.01 ± 7.78E-2	0.08 ± 0.02	0.05 ± 3.72E-3
Habitat.SE	16.76 ± 0.85	0.27 ± 0.02	-7.23 ± 0.28*
MAT	-1.23 ± 0.02***	-0.84 ± 1.25	-2.47 ± 0.28***
MAP			29.06 ± 6.68
MAP^2^	-0.04 ± 1.77E-03***	0.70 ± 0.22***	
Elevation:MAT	9. 23E-4 ± 2.24E-5**	5.26 ± 3.60	0.26 ± 0.03***
Elevation:Habitat.DE			0.35 ± 0.01
Elevation:Habitat.SE			0.57 ± 0.07
Elevation:elevation^2^	-7.96E-4 ± 6.49E-5*	-0.37 ± 0.06	
Elevation^2^:Habitat.DE			-53.4 ± 6.65*
Elevation^2^:Habitat.SE			5.42E-6 ± 8.04E-7
Elevation^2^:MAT	-0.07 ± 6.49E-3	-8.41 ± 4.13***	0.03 ± 6.67E-3***
Habitat.DE:MAT			-71.2 ± 7.82
Habitat.SE:MAT			28.46 ± 4.17*
MAT:MAP			34.74 ± 1.94***
Elevation:elevation^2^:MAT		2.10 ± 0.89**	
Elevation:Habitat.DE:MAT			-0.05 ± 1.94E-3*
Elevation:Habitat.SE:MAT			10.24 ± 2.29*
Elevation^2^:Habitat.DE:MAT			1.23E-5 ± 1.94E-6
Elevation^2^:Habitat.SE:MAT			-3.70E-3 ± 2.33E-4*

Hump-shaped diversity patterns have been reported for different groups of vascular plants [24], such as palms, Acanthaceae, Bromeliaceae and woody plants along tropical mountains [51–53]. Compared to previous findings, our results show the peak in α-diversity shifted towards higher, instead of mid-elevations [54]. One explanation for this pattern is that herbs are adapted to cold climates and had a success in temperate zones due to be annual and the production of underground structures (e.g. rhizomes and stolons) [55].

Lower elevations in Veracruz are subject to prolonged dry seasons (Table 2), which may limit α-diversity. With increasing elevation, precipitation increases, while temperatures and potential evapotranspiration decrease. Species richness of ferns has been reported to be positively related to humidity [56]. In our study, angiosperm richness peaked between 2500 m and 3100 m, where precipitation and the number of rainy days already decrease. However, the *Pinus*-*Quercus* forests at 2500 m are often subject to fog, whereas in *Pinus* forests (3100 m) light transmission to the forest floor is high [57], which likely increases the ground cover of angiosperms and thus also their diversity.

Surprisingly, forest-use intensity had no significant effect on α-diversity (Fig 3). The lack of a detectable net-change in α-diversity might indicate that the level of forest-use intensity is still relatively moderate; however, other life forms (e.g. trees, epiphytes and ferns) might show contrasting patterns. It is quite well documented that forest herbs profit from better light conditions in DE or SE. Newbold et al. [35], for example, found that the richness of vascular plant species can increase by 40% due to the conversion of old-growth forests to secondary vegetation, but more severe habitat conversion, e.g. from forest to intensive cropland, decreases species richness.

### Proportion of endemic species

**Fig 4:**
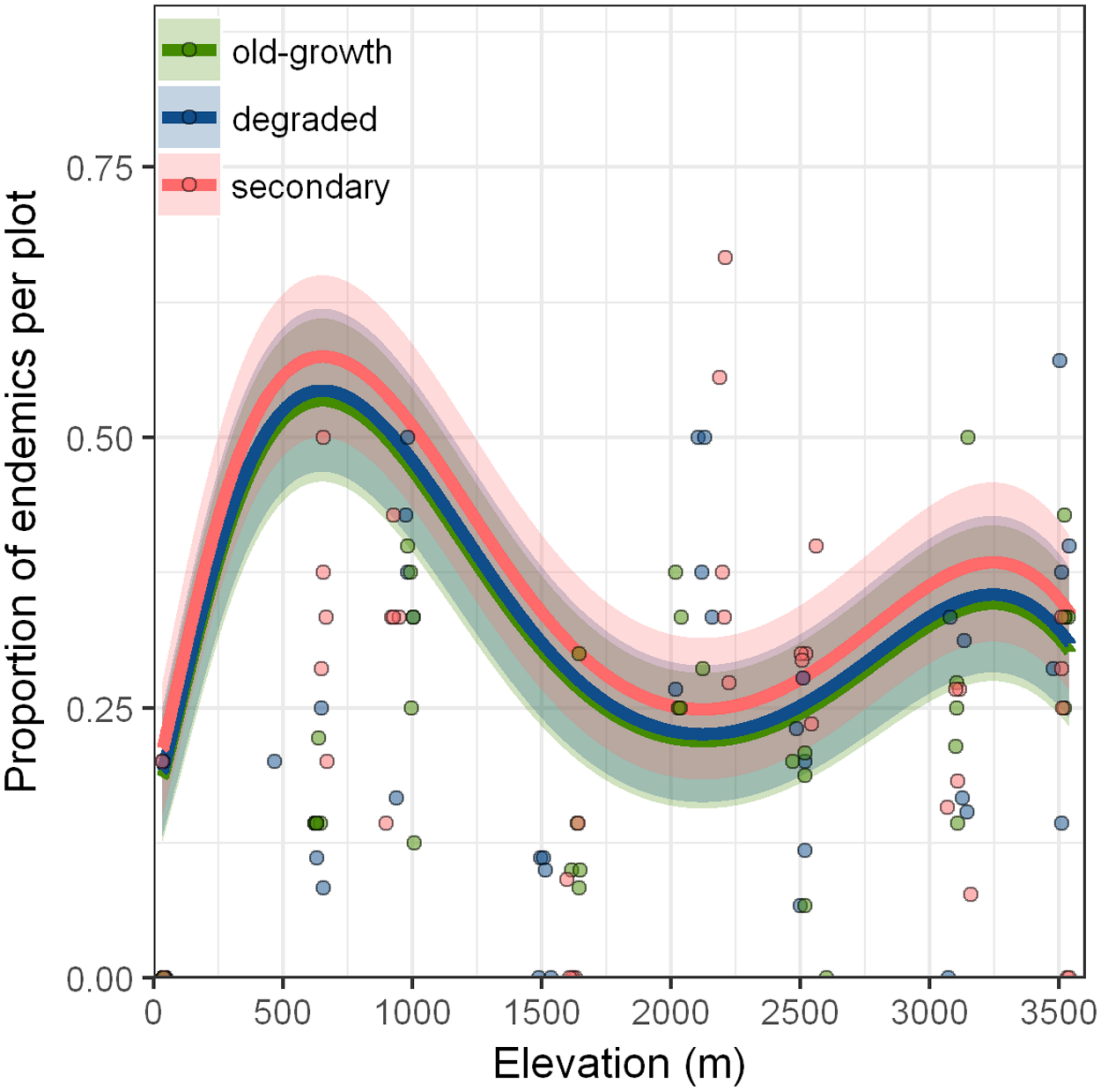
The proportion of endemic species of herbaceous angiosperms along gradients of elevation and forest-use intensity at the *Cofre de Perote*, central Veracruz, Mexico. We fit the lines from a GLM with a beta error family, the shaded area marks confidence intervals (CI = 1.96 times standard error). Difference to zero is not significant for the intercept (xy, OG at 50 m, p = 0.066) as well as for the effect of habitat (DE: p = 0.771, SE: p = 0.226). Observed proportion of endemic species on 20 m × 20 m plots along the elevational gradient for OG (green), DE (blue), and SE (red).

The best model for the proportion of endemic species was the one that included elevation, elevation^2^, habitat, MAT, MAP^2^ and the interaction between elevation and MAT, elevation and elevation^2^, elevation^2^ and MAT and a triple interaction with elevation, elevation^2^ and MAT (Fig 4, Table 3). The proportion of endemic species showed a non-linear pattern along the elevational gradient with the highest proportion at 500 m (Fig 4, Table 4). There is no effect of forest-use intensity on the proportion of endemic species, the lack of a pattern on the endemic species can be attributed to the adaptation of the endemic herbaceous plant's species of Mexico to several patterns of disturbance or the extinction of previous endemic species. This model of the proportion of endemic species had an *R*^2^ of 0.41.

### Beta and gamma diversity

In contrast to richness, forest-use intensity had marked effects on floristic composition, which was most visible when looking at habitat transitions (Table 5). Within-habitat β-diversity was generally lower (0.42–0.52) than between-habitat β-diversity (0.58–0.66). Unsurprisingly, we found the highest dissimilarity in the transition from OG to SE, whereas the most homogenous species pool was within OG. The high β-diversity between habitats indicated wider environmental differences [58] than the heterogeneity of plots within the same habitat type.

**Table 5:** Average effects of the habitat change. Mean β-diversity at every habitat transition, letters in superscript differences in groups after Tukey posthoc test (HSD = 0.138).

Habitat transition	β-diversity (1-S)
Old-growth to secondary	0.66^a^
Degraded to secondary	0.61^ab^
Old-growth to degraded	0.58^ab^
Degraded to degraded	0.52^bc^
Secondary to secondary	0.48^bc^
Old-growth to old-growth	0.42^c^

**Fig 5:**
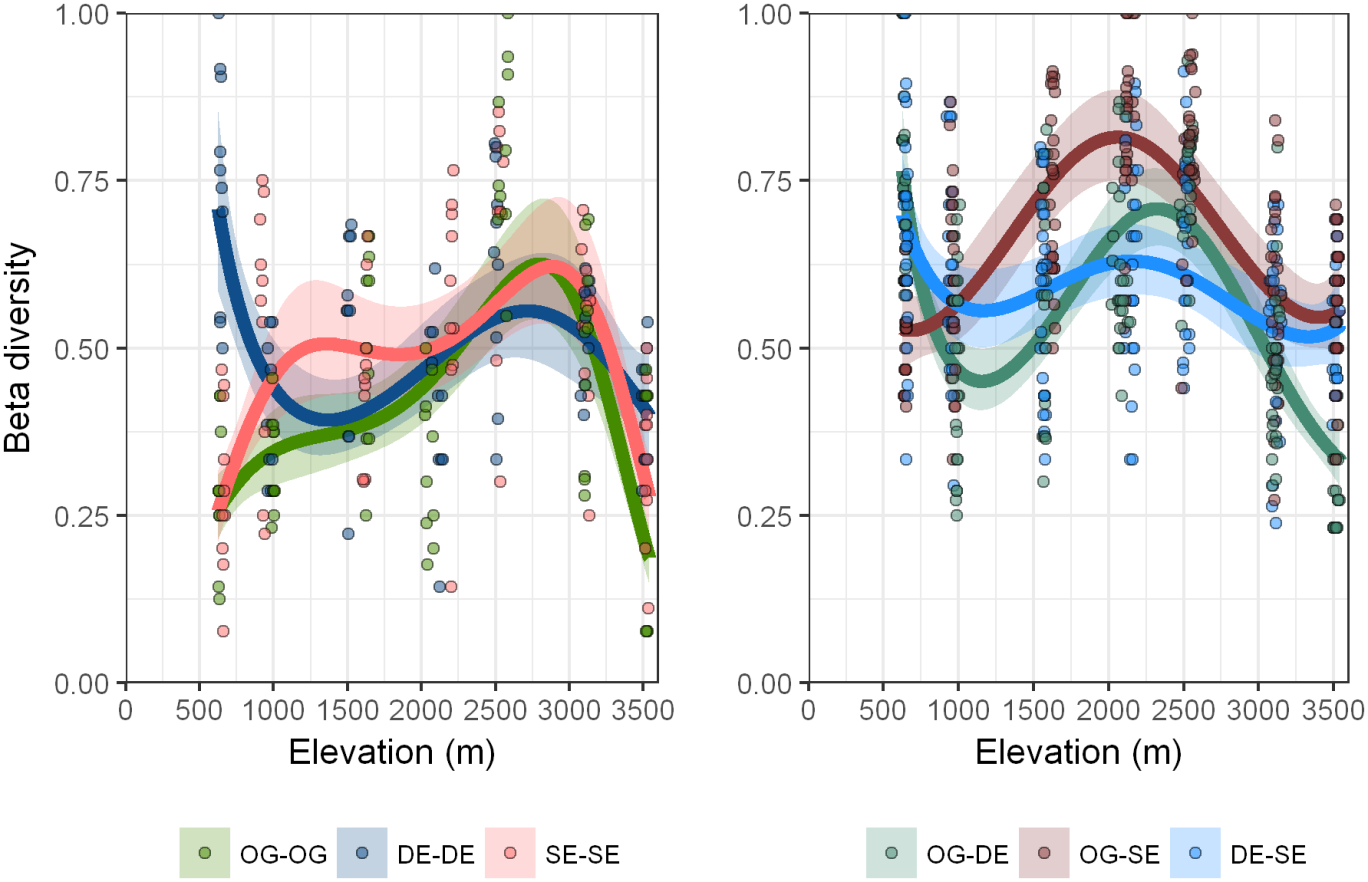
Compositional heterogeneity (as a measure for β-diversity) among different changes in forest habitats along the elevational gradient at the *Cofre de Perote*, central Veracruz, Mexico. Values are 1-Sørensen values as means across all plots. Shadows are standard errors computed by a GLM with log-normal error family.

The best model for β-diversity included the main effects of elevation, elevation^2^, change, MAT, MAP and interactions between elevation and change, elevation and MAT, elevation^2^ and change, elevation^2^ and MAT, change and MAT, MAT and MAP, as well as triple interactions among elevation, change, MAT and elevation^2^, change, MAT (Table 3). This model explains little variation (*R*^2^ = 0.49). There was a marked effect of elevation on β-diversity (Fig 5, Table 4). Within-habitat β-diversity showed a clear humped-shaped pattern for OG and SE with peaks between 2500 m and 3000 m. DE, however, had their highest β-diversity at 650 m with a subsequent decline. Obviously, DE at lower elevations exhibits a different response to environmental conditions compared to OG and SE. Degradation may lead to a higher heterogeneity of environmental conditions and, consequently, offer diverse niches triggering community differentiation [59]. Beta-diversity between-habitats was generally high but varied with the type of habitat transition. In the transition from OG to SE, there was a turnover of *c*. 50% of the species at both extremes of the elevational gradient. Between 1500 m and 2500 m, however, 75% of the species were different when comparing OG to SE. Habitat transitions related to degradation (OG-DE, DE-SE) showed highest β-diversity at 650 m and 2500 m and declined with increasing elevation. Endemic species contribute to this pattern only at 650 m where we found their peak (Fig 4). Especially at 3100 m and 3500 m, the change in species composition with the transition from OG to DE was relatively low. This indicates that present environmental conditions favour a spectrum of adapted species [60], which thrive regardless of the habitat type. Above 3100 m there are fewer species, which are adapted to extreme climate events, such as days below 0°C, lower temperature and precipitation (Table 1).

**Fig 6:**
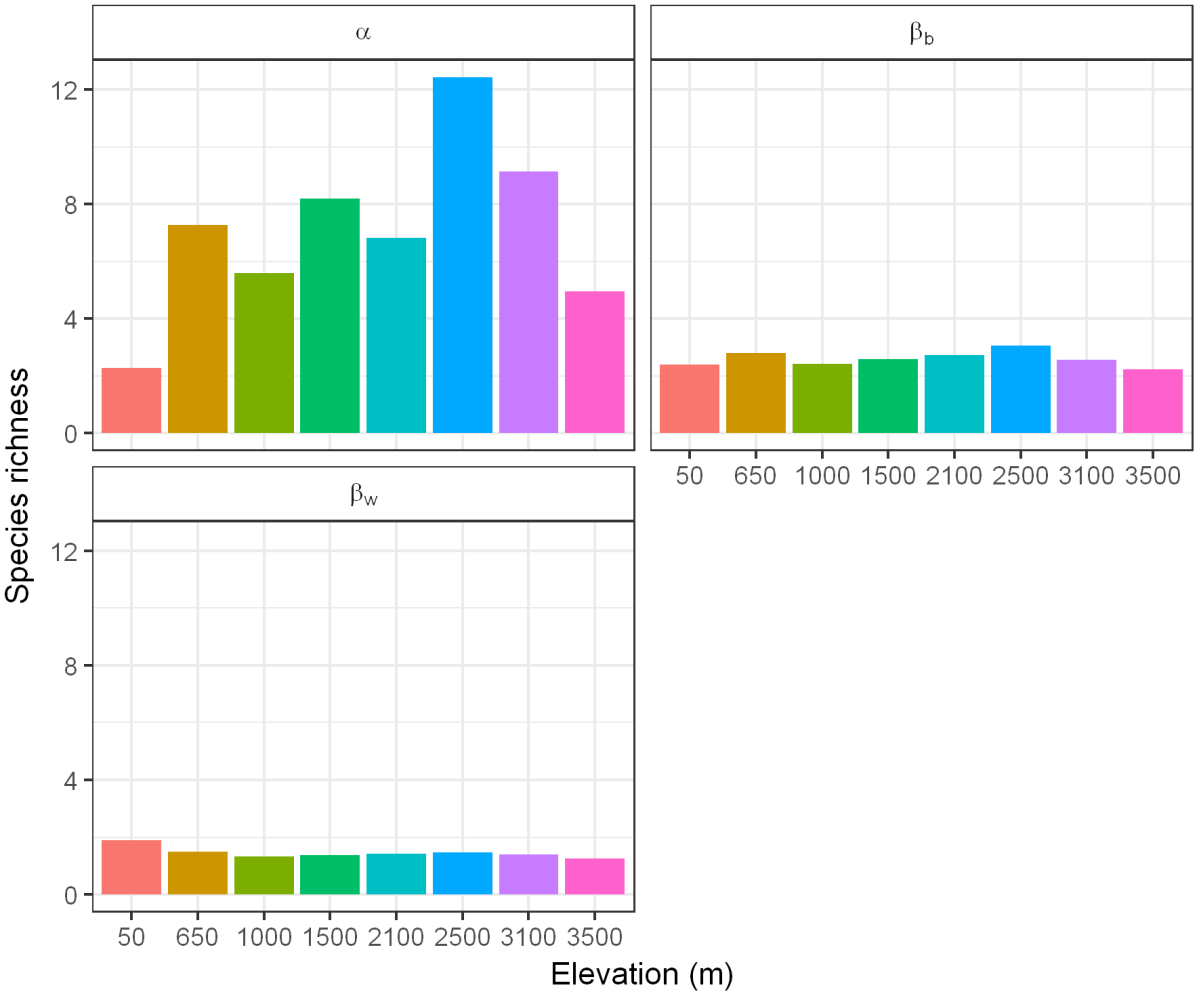
Multiplicative diversity partitioning. Multiplicative version isolating pure relative differentiation (e.g. β is independent of α). Here, the number of distinct units of the lower level of partition can explain the relative β-diversity and multiply to equal γ-diversity.

We revealed the relative contribution of relative α, relative within-habitat β, and relative between-habitats β-diversity to γ using the multiplicative partitioning approach (Fig 6). The contribution of relative α-diversity was only between 3 and 13 (Fig 6). Diverse forest habitats support high species diversity and lead to high relative β-diversity between-habitats (β_b_) confirming the importance of these habitats. We found a hump-shaped pattern of relative α-diversity (number of species per plot) with a peak at 2500 m and decreases towards the extremes. There was also a hump-shaped pattern of the relative contribution of between-habitat β-diversity (difference in species composition between plots within a habitat) and a decline of within-habitat β-diversity with elevation. The adaptation of some herbs to cold climates could increase of relative α-diversity at higher elevations [55], which is similar to the pattern found by Cicuzza et al. [34] concerning the effect of forest structure (e.g. more open canopies) [61]. Yang et al. [62] also found the pattern of relative β-diversity, which was explained by changes in climatic variables as elevation-related vegetation zones reflect climatic zones. This is observable in our results as the most remarkable changes in values of relative β_b_-diversity occurred between different climatic zones (Fig 6), which demonstrates the effects of an elevation-related climate gradient on relative β_b_-diversity patterns [62]. The increasingly stronger control of climate over other environmental factors (e.g. soil factors) and the decrease of forest heterogeneity at higher elevations may explain the decline in relative β_w_-diversity which is consistent with the results of Akhtar and Bergmeier [63], who worked with herbs in the mountains of Northern Pakistan.

The most remarkable effect of forest-use intensity was on β-diversity and it was highest between 2100 m and 2500 m (Fig 5). It has been reported that forest-use intensity decreases β-diversity due to the propagation of exotic and opportunist species that can lead to a ‘biotic homogenization’ [64]. Beta-diversity was, on average, in our sample of landscapes, lower within habitats than between habitats (Table 5 and Figs 5 and 6). Additionally, the α-diversity in our study did not change, but the dissimilarity between habitats was high (0.594 to 0.665) (Table 5).

Here, we argue that all habitats and even anthropogenic habitats contribute to the herbaceous angiosperm richness in our study region with seven species endemic to Mexico and seven native species exclusive from these habitats [36]. It is, of course, important to note that our study does not include spatially weighted information about the abundance of different forest habitats. Pressure on the primary or OG forest is often higher than their ability to regenerate. Therefore, there is the risk that OG or even DE will be converted into SE [65]. Although different successional stages of SE may also harbor high species richness [66], a homogenization of habitats will consequently lead to species homogenization by decreasing β-diversity.

The most vulnerable location is the *Pinus*-*Quercus* forest at 2500 m because high β_b_-diversity implies a loss of many OG species during forest degradation. This means that conversion of a certain area increases the chance that a unique flora is changed in composition and invasive species appear. In addition, this elevation contains the largest number of endemic species compared to the rest of locations [36], leading to increased vulnerability. Therefore, the *Pinus*-*Quercus* forest at 2500 m should be considered as a priority for conservation, especially because according to Mittermeier et al. [67] this vegetation type in Mexico has the lowest levels of protection [68]. For the herbaceous angiosperm group, however, it seems that a well-designed management plan instead of pure conservation would be beneficial because high habitat heterogeneity is required to achieve high species richness.

**Fig 7:**
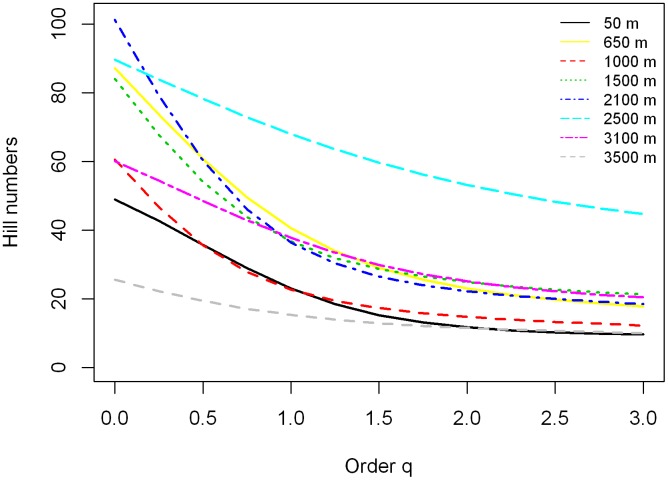
Asymptotic analysis: The asymptotic diversity profile as a function of order *q* based on the adjusted data. The estimated diversity profiles for *q* between 0 and 3 based on the sample frequency counts of herbaceous angiosperms from eight locations.

For the asymptotic analysis, we plotted the estimated asymptotic diversity profiles when *q* is between 0 and 3 (Fig 7). The empirical diversities imply that the eight locations differ in each of the *q* orders. In contrast, the plots in Fig 7 reveal that for the asymptotic *q* = 0 the 2100 m location is substantially more diverse than the other locations. However, for the Shannon diversity (*q* = 1), the 2500 m location is substantially more diverse than the other locations. A similar conclusion is also valid for the Simpson diversity (*q* = 2), confirming our other analyses (Figs 3, 5 and 6).

## Conclusions

In order to understand how forest-use intensity and elevation affect species diversity and community composition of herbs, we focused on the three components of diversity (α, β, and γ) that allowed us to understand the different patterns at the landscape level. We did not find significant differences in α-diversity among the three forest systems, a finding that is not in accordance with previous studies [1,4,6,15]. However, forest-use intensity affected the floristic composition, which varied markedly between habitats. The most important component driving γ-diversity was the β-diversity between habitats. Thus, different forest-use intensities, which coexist, increased the species richness in the landscape.

Some elevations, and especially the location at 2500 m, were evidently vulnerable, whereas species richness still depends on a certain degree of forest-use intensity. Our findings clearly showed that OG at mid-elevations contributed more to regional diversity than DE. In the case of herbaceous angiosperms, sustainable forest management, such as forest certification instead of strict protection may be a good way to conserve herbaceous forest plants in the region. Forest protection systems should consider the important influence of β-diversity to regional species richness rather than put emphasis completely on the protection at local scale (α) diversity.

## Supporting information

S1 File Presence/absence data of the herbaceous angiosperms found at the plots sampled along the elevational gradient at the Cofre de Perote, central Veracruz, Mexico. (CSV)
